# Cooling Performance of a Novel Circulatory Flow Concentric Multi-Channel Heat Sink with Nanofluids

**DOI:** 10.3390/nano10040647

**Published:** 2020-03-31

**Authors:** Ravindra Jilte, Mohammad H. Ahmadi, Ravinder Kumar, Vilas Kalamkar, Amirhosein Mosavi

**Affiliations:** 1 Department of Mechanical Engineering; Lovely Professional University, Phagwara, Punjab-14411, India; rdjilte@gmail.com (R.J.); rav.chauhan@yahoo.co.in (R.K.); 2 Faculty of Mechanical Engineering, Shahrood University of Technology, POB- Shahrood 3619995161, Iran; 3 Department of Mechanical Engineering, Visvesvaraya National Institute of Technology, Nagpur 440010, India; vilas.kalamkar@gmail.com; 4 Kalman Kando Faculty of Electrical Engineering, Obuda University, 1034 Budapest, Hungary; 5 Institute of Structural Mechanics (ISM), Bauhaus-Universität Weimar, 99423 Weimar, Germany; 6 Thuringian Institute of Sustainability and Climate Protection, 07743 Jena, Germany; 7 Faculty of Civil Engineering, Technische Universität Dresden, 01069 Dresden, Germany

**Keywords:** heat sink, mini-channels, liquid cooling, nanofluid, cooling performance

## Abstract

Heat rejection from electronic devices such as processors necessitates a high heat removal rate. The present study focuses on liquid-cooled novel heat sink geometry made from four channels (width 4 mm and depth 3.5 mm) configured in a concentric shape with alternate flow passages (slot of 3 mm gap). In this study, the cooling performance of the heat sink was tested under simulated controlled conditions.The lower bottom surface of the heat sink was heated at a constant heat flux condition based on dissipated power of 50 W and 70 W. The computations were carried out for different volume fractions of nanoparticles, namely 0.5% to 5%, and water as base fluid at a flow rate of 30 to 180 mL/min. The results showed a higher rate of heat rejection from the nanofluid cooled heat sink compared with water. The enhancement in performance was analyzed with the help of a temperature difference of nanofluid outlet temperature and water outlet temperature under similar operating conditions. The enhancement was ~2% for 0.5% volume fraction nanofluids and ~17% for a 5% volume fraction.

## 1. Introduction

Miniaturization of electronic devices faces heat rejection problems. Advancement in fabricating these devices has resulted in them becoming smaller in size. Such devices produce a large heat generation in a smaller volume. It necessitates an effective cooling arrangement for maintaining the operating temperature within a safe range [1]. Uses of heat sinks with micro/mini-channels have emerged as a feasible solution, which was first proposed by Tukerman and Pease [2]. Among the possible working fluids investigated, air cooling is unable to meet the increasing demand for high heat removal rates. Electronic and IT applications demand a compact, more efficient, and an adequately designed effective cooling system that is capable of sustainable longevity. Generally, these electronics systems are cooled with either air or liquid (water, glycol, etc.) [3]. Microchannel liquid cooling can be advantageous [4].

Many investigations have reported on hydraulic and thermal behavior as well as heat transfer augmentations. The different heat-transfer augmented techniques in micro/mini-channels for single-phase cooling devices were reviewed by Steinke and Kandlikar [5].An investigation was performed on a water-cooled offset strip fin enhanced microchannel heat exchanger and this was shown to perform better when compared with straight continuous channel walls [6]. Khameneh et al. [7] carried out a numerical study on laminar flow and forced convective heat transfer in water-cooled rectangular-shaped microchannel sections that had specific hydraulic diameters and distinct geometric configurations. Aspect ratio and hydraulic diameter affected the heat transfer rate of microchannels. Hasan et al. [8] analyzed the performance of a microchannel heat exchanger and presented results which took into consideration the different shapes and sizes of the channels. Xie et al. [4] performed a numerical study on mini-channel heatsinks subjected to a constant heat flux wall condition. In narrow and deep channels, the heat transfer performance was improved with a relatively high-pressure drop. Jiang and Ruina [9] reported that, in mini-fin structures, the convective heat transfer coefficient increased 9–21-fold for water and 12–38-fold for air, compared to the empty plate channel. Ahmed [10] conducted a numerical study on grooved microchannel heatsinks to analyze the effect of geometrical specifications on laminar convective heat transfer. Trapezoidal grooved microchannel heatsinks (MCHSs) have the optimum thermal design compared to rectangular and triangular grooved MCHSs. Choi et al. [11] conducted numerical analysis on microchannel water blocks with pass variations. The heat transfer rate accompanied by higher values of pressure drop were observed in two-pass samples. Jajja et al. [12] experimentally investigated the influence of fin spacing in different heat sinks for effective thermal management. Liu and Jianlin [13] numerically analyzed the fluid flow and thermal characteristics of mini-channel heatsinks with non-uniform inlets. The total thermal resistance of the mini-channel heat sink was reduced by 9.9% to 13.1% using non-uniform baffles. Aliabadi et al. [14] experimentally studied the cooling performance of a sinusoidal wavy mini-channel heat sink and examined the effect of geometrical parameters and working fluids and observed the effective thermal performance compared to a straight mini-channel heatsink. Lee et al. [15] suggested that a continuum-based approach can be applied to estimate the heat transfer in microchannels. The use of a serpentine microchannel (with a square cross-section) to characterize slug flow behavior has been reported by Cairone et al. [16]. Under critical heat flux conditions, heat rejection from heat sink utilizing water as the heat transfer media can be further enhanced with nanofluids [17].

Many studies have been carried out citing nanofluid usage in heat transfer enhancement [18,19,20]. Among these, Choi et al. [21] introduced the concept of nanofluid and presented that thermal conductivity of base fluid can be increased with the addition of nanoparticles of sizes less than 100 nm [22]. The use of such nanofluids in mini-channel heat sinks (MCHSs) has been reported in some studies. Ho et al. [23] carried out forced convective cooling of MCHSs with Al_2_O_3_–water nanofluid and found that it enhanced heat transfer. Koo and Kleinstreuer [24] suggested nanofluid selection should be based on higher Prandtl numbers, high-volume concentrations of nanoparticles, and a high aspect ratio of microchannels to avoid nanoparticle accumulation. Jang and Choi [25] found that MCHS performance was enhanced by 10% for a water-diamond nanofluid compared with pure water. Ijam and Saidur [26] analytically investigated the effect of nanoparticle concentration and Reynolds number in MCHSs.

Based on the studies above, most of the investigations were carried out on the characteristics of heat transfer and fluid flow in circular or rectangular straight mini-channel heat sinks. Only a few experimental or numerical studies are currently available with regards to spiral or concentric channels, but there is no research currently available which has reported on a multi-circular mini-channel heat sink. In this work, thermal performance of a concentric channel heat sink with an alternate slot for the fluid flow is presented.

## 2. Circulatory Flow Multi-Channel Heat Sink

**Figure 1:**
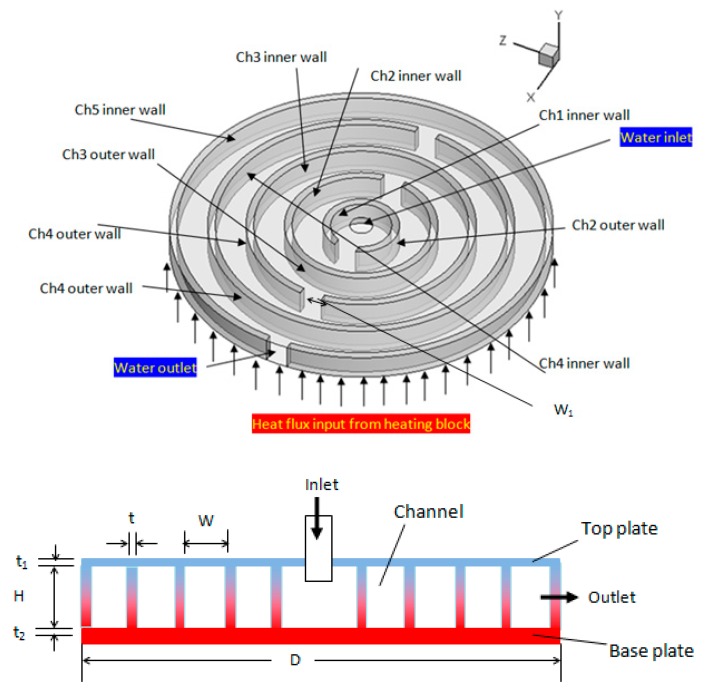
Proposed circulatory flow multi-channel heat sink.

A heat sink was modified to create a circulatory flow of cooling media. It contained four concentric channels (width 4 mm and depth 3.5 mm) with alternate opening slots (Figure 1). The cooling fluid was fed centrally to the heat sink to facilitate higher heat removal. An electronic device such as a processor generates more heat at the center, whereas peripheral portions or parts can dissipate heat to the surroundings. Cooling liquid from the inlet pipe entered centrally, and the flow bifurcated after passing through the first slot or opening. The liquid then flowed through the first channel again and then bifurcated after it passed through the second slot. The outlet for circulating liquid was provided at the outer periphery. The interior view of the heat sink (as shown in Figure 1) was made of copper and sealed on top with a copper plate. This top cover was provided with an inlet pipe which allowed the flow of inlet water through the channel. The geometrical dimensions of concentric circulatory flow heat sink are listed in Table 1.

**Figure 2:**
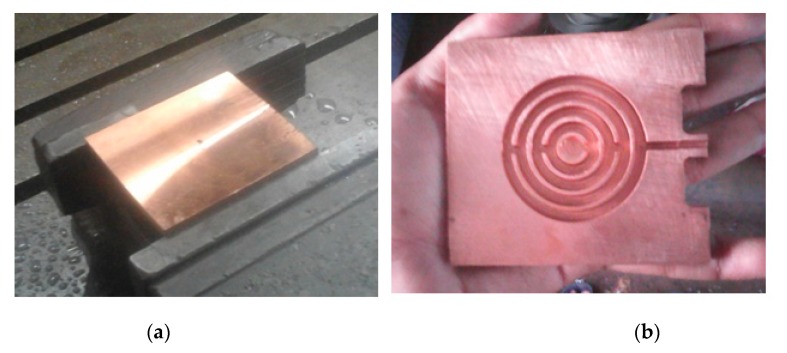
Heat sink during fabrication: (**a**) copper plate; (**b**) interior view of heat sink with channels.

**Figure 3:**
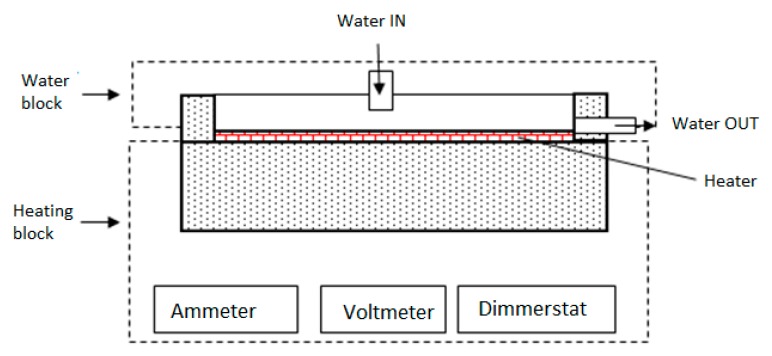
Arrangement showing water and heating.

The copper plate of required dimensions is used as heat sink material (Figure 2a). Due to the miniature size of the heat sink, it is fabricated using CNC machining (Figure 2b). In this study, cooling performance of the heat sink has tested under simulated controlled conditions. The electronic device that dissipates heat and needs cooling is replaced with equivalent heating block illustrated in Figure 3. The heating block majorly comprised of nichrome plate heaters placed directly beneath the lower surface of the heat sink. The amount of heat flow through this heater can regulated through different knob settings of the dimmerstat. The wattage of the electrical input is computed based on measured value of current and voltage. The heating block is provided with insulation to avoid heat losses to the surroundings. The experimental set up is assisted with instruments for measuring surface temperature of heat sink, inlet and outlet cooling liquid temperature, mass flow measurement, voltmeter and ammeter for voltage and current measurement, dimmerstat for wattage control, and control valves. Set up is created for continuous operation under steady-state conditions with the help of flow from water tank.

During the experiment, a constant input power of 50 W was supplied to the heating block. The bottom wall temperature of the heating block was recorded after the attainment of steady-state conditions. Experiments were conducted at different flow rates. A set of three trials were conducted to check reproducibility of the experimental data.

## 3. Numerical Modelling

### 3.1. Geometry

**Figure 4:**
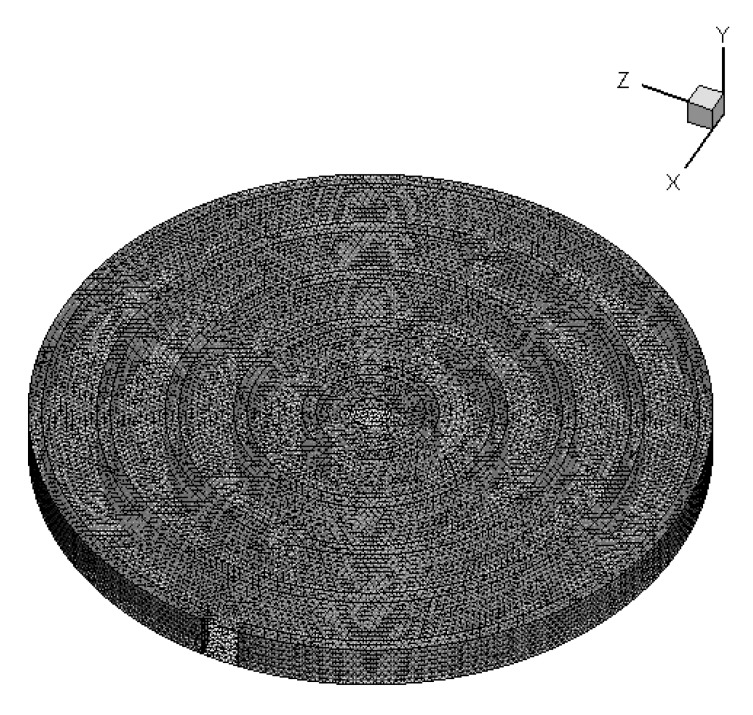
Three-dimensional geometry meshed model.

The present numerical work aimed at providing an insight into thermal performance throughout the flow direction. The limitation of installing temperature sensors in a mini-channel restricts the provision of any details on channel-to-channel heat removal. As a result, the three-dimensional modeling and meshing were created using software package GAMBIT 2.3.16 as shown in Figure 4. CFD Software package FLUENT 6.3.26 was used to perform numerical computations.

### 3.2. Nanofluid Properties

In this investigation, Al_2_O_3_ nanoparticles were used due to their ability to enhance heat transfer. The thermophysical properties of base fluid (water) and nanoparticles (Al_2_O_3_) are listed in Table 2.

For a lesser volume fraction of nanoparticles in the base fluid, the nanofluid can be treated as a single-phase, homogenous liquid. Nanofluid properties depend on the percentage of nanoparticles used in the base fluid. The thermophysical properties of nanofluids are calculated by the following equations:

Density [27,28,29]:

(1) $$\rho_{nf}=\left(1-\emptyset \right)\rho_{b}+\emptyset \rho_{p}$$

Viscosity, Einstein’sequation [30]:

(2) $$\mu_{nf}=\mu_{nf}\left(1+2.5\emptyset \right)$$

Thermal conductivity [31,32,33]:

(3) $$\;k_{nf}=\left[\frac{k_{p}+2k_{bf}+2\left(k_{p}-k_{bf}\right){\left(1+\beta \right)}^{3}\emptyset }{k_{p}+2k_{bf}-2\left(k_{p}-k_{bf}\right){\left(1+\beta \right)}^{3}\emptyset }\right]k_{bf}$$

where β is taken as 0.1 [34].

Specific heat [34,35]:

(4) $${\left(\rho C_{p}\right)}_{nf}=\left(1-\emptyset \right){\left(\rho C_{p}\right)}_{bf}+\emptyset {\left(\rho C_{p}\right)}_{p}$$

where *ϕ* denotes volume fraction of nanoparticles and subscript *nf, bf, p* denotes nanofluid, basefluid and particle, respectively.

### 3.3. Governing Equation and Boundary Conditions

The nanofluid was taken as single-phase fluid and subjected to the following flow assumptions: steady state, incompressible, laminar, and constant properties. The governing equations for solving flow conditions are given below [36]: Continuity equation:

(5) ∇(ρv)=0

Momentum equation:

(6) ∇(ρvv)=−∇P+∇(μ∇v)

Energy equation for the fluid:

(7) $$\nabla \left(\rho vC_{p}T\right)=\nabla \left(k\nabla T\right)$$

Energy equation for the solid wall:

(8) ∇(k∇T)=0

Boundary conditions:

Heat sink walls are subjected to no-slip boundary conditions, whereas the inlet and the outlet of the domain are given as velocity inlet and pressure outlet, respectively. As shown in Figure 1, the lower bottom surface of the heat sink was heated at a constant heat flux condition based on dissipated power of 50 and 70 W.

### 3.4. Numerical Scheme and Validation

**Figure 5:**
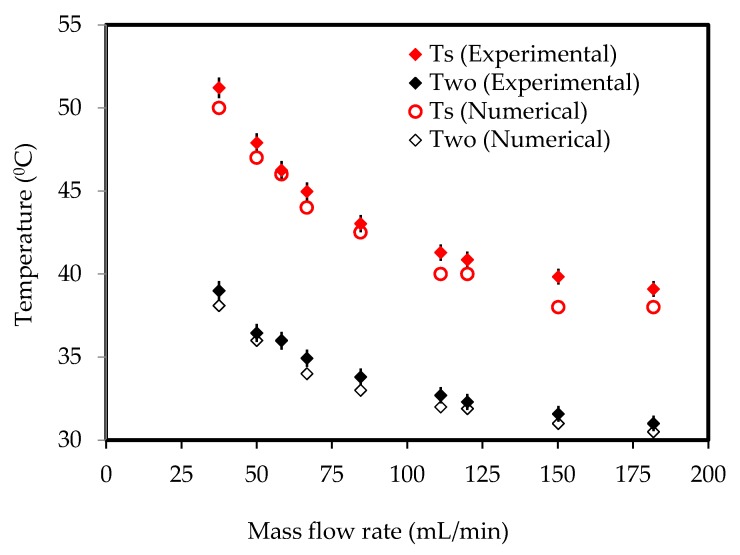
Validation of the numerical model adopted for the analysis.

The control volume-based approach was adopted for solving governing equations (Equations (6)–(9). The SIMPLE algorithm was chosen for pressure-velocity coupling. A steady-state laminar model was used for the analysis, and the convergence criteria for residuals of continuity and velocity equations were of the order of 10^−6^. For the energy equation, they were of the order of 10^−9^. The results were obtained once the solutions were converged. Figure 4 shows the plot of surface temperature and water outlet temperature measured experimentally as described in Section 2. The area-weighted surface and water outlet temperatures were obtained at similar flow conditions and were plotted in Figure 5. It was observed that numerical results were in good agreement with the experimental data and, therefore, the present numerical scheme should be adopted for further study to give insights into heat transfer.

## 4. Discussion

The cooling performance of the heat sink was carried out at heat generation rates of 50 and 70 W. Under each heating condition, heat dissipation was computed out for four different flow rates at 30 to 180 mL/min. The computational results are presented below.

### 4.1. Flow Field in Heat Sink

**Figure 6:**
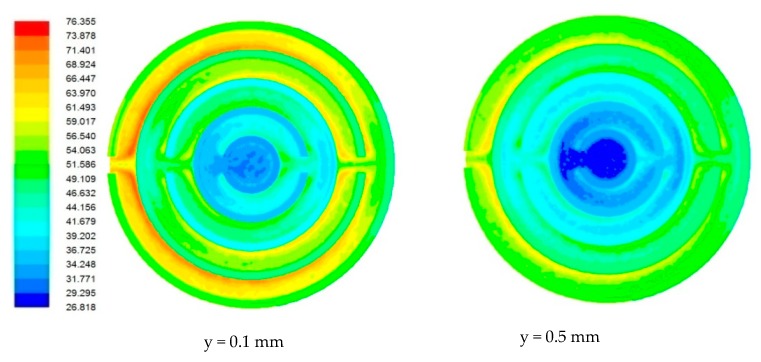
Contours of the temperature field in the heat sink for a flow rate of 30 mL/min.

**Figure 7:**
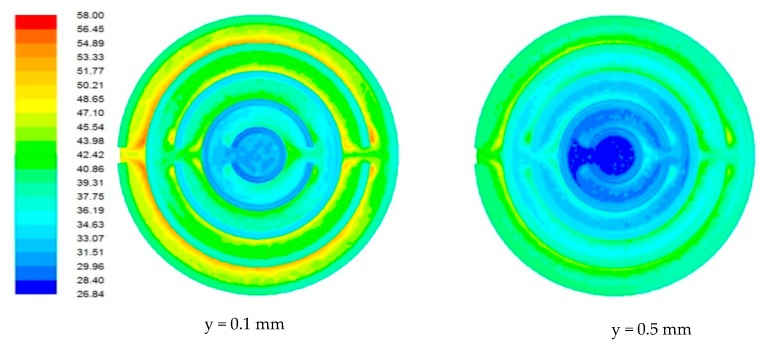
Contours of the temperature field in the heat sink for a flow rate of 60 mL/min.

**Figure 8:**
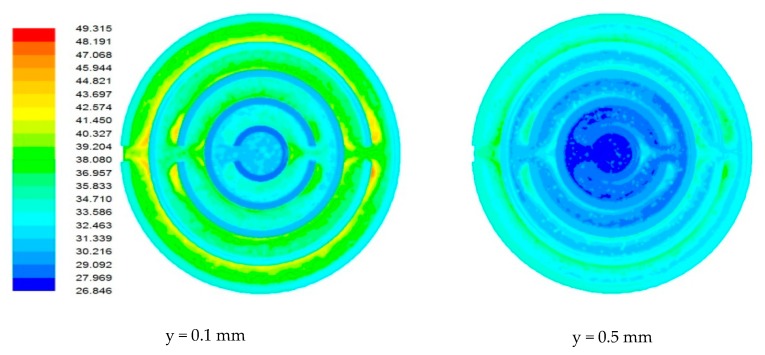
Contours of the temperature field in the heat sink for a flow rate of 120 mL/min.

**Figure 9:**
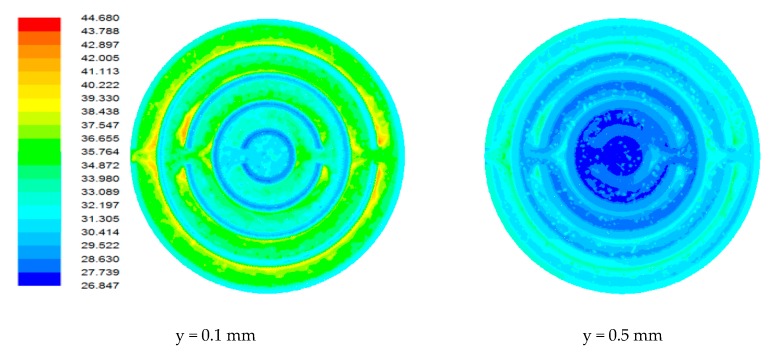
Contours of the temperature field in the heat sink for a flow rate of 180 mL/min.

Due to the miniature size involved in the mini-/microchannel heat sink and difficulty in sensor placement, flow field and temperature distribution were difficult to measure experimentally. The post-processing tools available in computational software allowed detailed flow-field analysis. Figure 6, Figure 7, Figure 8 and Figure 9 show sample contours of temperature for a heat generation rate of 50W. Temperature flow fields were analyzed at depths of 0.1 mm and 0.5 mm from the base surface of the heat sink. It can be observed that the temperature of the cooling fluid increased along the flow direction. At the center of the heat sink, cooling fluid was at its lowest temperature. It collected heat from the heat sink as it passed through different channels. It was observed that the fluid layers adjacent to the solid bottom wall collected heat by conduction, whereas other fluid layers above underwent forced convective heat transfer. The temperature fields developed in the heat sink were also analyzed at different flow rates. It was observed that lower flow rates were not adequate in maintaining a lower heat sink temperature. At a lower flow rate, there could be potential danger of obtaining a temperature that is beyond that of safe operation. The maximum local temperature (~77°C) attained at a low flow rate (Figure 6) can be reduced to 58°C (Figure 7), 49.31°C (Figure 8), and 44.61 °C (Figure 9) for flow rates of 60, 120, and 180 mL/min, respectively.

### 4.2. Heat Transfer Enhancement with Nanofluids

**Figure 10:**
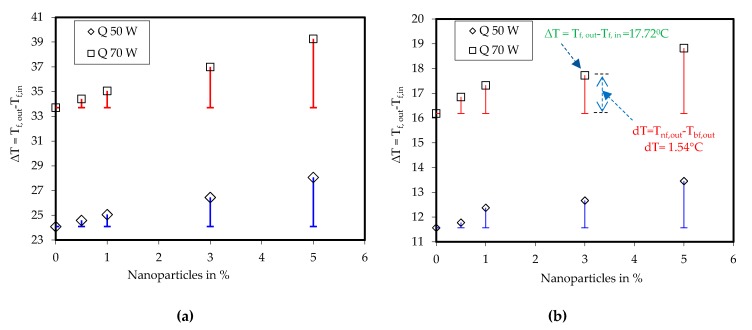
Temperature difference between liquid outlet and liquid inlet and its comparison with base fluid at (**a**) 30 mL/min, (**b**) 60 mL/min.

**Figure 11:**
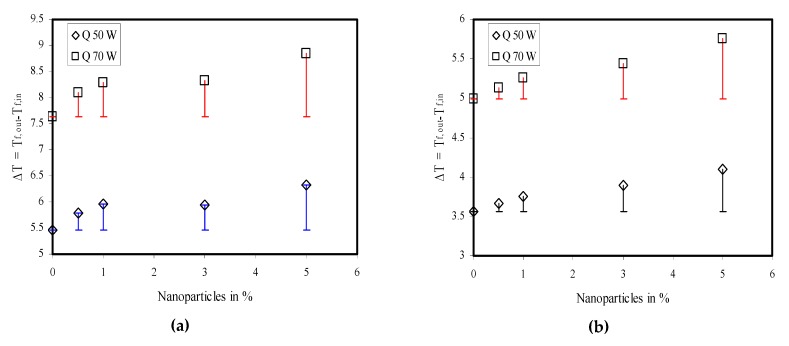
Temperature difference between liquid outlet and liquid inlet and its comparison with base fluid at (**a**) 120 mL/min, (**b**) 180 mL/min.

The fluid outlet temperature was considered to analyze the device’s cooling performance. Initially, the heat sink was cooled with water for different flow rates and taken as base for its comparison with nanofluid. The computations were carried out for different volume fractions of nanoparticles, namely 0.5%, 1%, 3%, and 5%. The following two temperature differences were compared:

(9) $$\begin{array}{c} \Delta T=T_{f,out}-T_{f,in}\\ dT=T_{nf,out}-T_{bf,out} \end{array}\}$$

where *T_f,out_* is the liquid outlet temperature, *T_f,in_* is the liquid inlet temperature, *T_nf,out_* is the nanofluid outlet temperature, and *T*_bf,out_ is the water outlet temperature. The results are plotted in Figure 10 and Figure 11.

The computed data were analogous to the experimental observation shown earlier in Figure 5. The liquid outlet temperature was reduced when the liquid flow rate was increased and, therefore, ∆*T* also reduced the constant liquid inlet temperature. At a low mass flow rate of 30 mL/min and when Q = 50 W, ∆*T* was approximately 24 °C. Under this condition, the nanoparticle addition in the base fluid enhanced ∆*T*. The use of nanoparticles increased d*T* by 0.5 °C to ~3.97 °C for nanoparticles volume concentrations of 0.5% and 5%.Similarly, for the same flow rate of 30 mL/min and Q = 70 W, ∆*T* was approximately 34 °C. The nanofluid raised d*T* by 0.7 °C to ~5.55 °C for nanoparticles volume concentrations of 0.5% and 5%. The other computational results are plotted in Figure 11.

## 5. Conclusions

In the present work, modified heat sink geometry was introduced for heat dissipation from electronic devices. The cooling performance of the proposed heat sink was compared with Al_2_O_3_–water nanofluids. The conclusion can be summarized as follows:

(1) The maximum local temperature (~77 °C) attained at a low flow rate (30 mL/min) can be reduced to 58, 49.31, and 44.61 °C for flow rates of 60, 120, and 180 mL/min, respectively.
(2) At a lower mass flow rate of 30 mL/min and Q = 50 W, the temperature difference between water outlet temperature and water inlet temperature was approximately 24 °C. This reduced to ~4 °C as the mass flow was increased to 180 mL/min.
(3) A higher rate of heat generation of around 70 W produced a water outlet temperature of ~34 °C for a water flow rate of 30 mL/min. This was reduced to ~5 °C when the water flow was increased.
(4) Heat rejection rate enhanced with nanofluid usage. The enhancement was calculatedby measuring the temperature difference of nanofluid outlet temperature and water outlet temperature under similar operating conditions. The enhancement was ~2% for 0.5% volume fraction nanofluids to ~17% for a 5% volume fraction.

## Figures and Tables

**Table 1:** Specifications of the heat sink.

Parameters	Size, mm
Diameter of heat sink (D)	50
Height of channel (H)	3.5
Width of channel (W)	4
Thickness of channel wall (t)	1
Thickness of heat sink base plate (t_2_)	2
Thickness of heat sink cover plate (t_1_)	1
Total height of heat sink (H + t_1_ + t_2_)	5.5
Flow passage slot for the water to flow (W_1_)	3
Dimensions of the water outlet duct (W_1_× H)	3 × 3.5
Width of the slots (passage)	3

**Table 2:** Properties of base fluid and nanoparticles.

Properties	Pure Water	Alumina (Al_2_O_3_)
Mass density, kg/m^3^	995.81	3880
Specific heat, J/kgK	4178	765
Thermal conductivity, W/mK	0.6172	40
Viscosity, kg/ms	0.0008034	−
